# Afferent and Efferent Visual Markers of Alzheimer’s Disease: A Review and Update in Early Stage Disease

**DOI:** 10.3389/fnagi.2020.572337

**Published:** 2020-09-11

**Authors:** Shirley Z. Wu, Arjun V. Masurkar, Laura J. Balcer

**Affiliations:** ^1^Department of Neurology, New York University Grossman School of Medicine, New York, NY, United States; ^2^Department of Ophthalmology, New York University Grossman School of Medicine, New York, NY, United States; ^3^Department of Population Health, New York University Grossman School of Medicine, New York, NY, United States

**Keywords:** Alzheimer’s disease, mild cognitive impairment, visual biomarkers, afferent visual system, efferent visual system, optical coherence tomography, saccadic eye movement, pupillometry

## Abstract

Vision, which requires extensive neural involvement, is often impaired in Alzheimer’s disease (AD). Over the last few decades, accumulating evidence has shown that various visual functions and structures are compromised in Alzheimer’s dementia and when measured can detect those with dementia from those with normal aging. These visual changes involve both the afferent and efferent parts of the visual system, which correspond to the sensory and eye movement aspects of vision, respectively. There are fewer, but a growing number of studies, that focus on the detection of predementia stages. Visual biomarkers that detect these stages are paramount in the development of successful disease-modifying therapies by identifying appropriate research participants and in identifying those who would receive future therapies. This review provides a summary and update on common afferent and efferent visual markers of AD with a focus on mild cognitive impairment (MCI) and preclinical disease detection. We further propose future directions in this area. Given the ease of performing visual tests, the accessibility of the eye, and advances in ocular technology, visual measures have the potential to be effective, practical, and non-invasive biomarkers of AD.

## Introduction

Alzheimer’s disease (AD), the most common cause of dementia worldwide (McKhann et al., 2011), is a growing public health issue estimated to affect 13.8 million Americans by 2050 (Alzheimer’s Disease Facts and Figures, 2019). The pathophysiology of AD involves brain deposition of extracellular amyloid-beta plaques and intracellular tau neurofibrillary tangles, which lead to neuronal degeneration and subsequent cognitive impairment (Murphy and LeVine, 2010). AD is diagnosed by clinical and neuropsychiatric assessments complemented by neuroimaging (McKhann et al., 2011). Since it is a clinical diagnosis, individuals are often diagnosed at later stages when symptomatic. However, biomarkers of AD pathophysiology can precede clinical symptoms by many years (Fagan et al., 2007; Jack et al., 2009; Roe et al., 2013; Petersen et al., 2016; Betthauser et al., 2020), making it possible for earlier detection in predementia stages. The progression to AD dementia is divided into three broad stages. In the preclinical stage, individuals are cognitively and functionally normal on a standardized assessment but have biomarkers of AD pathology on a molecular or imaging level, and may be at-risk for future decline. As such, subjective cognitive decline in normal individuals has been proposed to be preclinical AD given its association with imaging and cerebrospinal fluid AD biomarkers and higher rates of future objective decline compared to those without subjective concern (Jack et al., 2018; Jessen et al., 2020). Mild cognitive impairment (MCI) due to AD is considered the prodromal stage, which includes individuals with objectively measurable cognitive deficits but with intact or only minimally impaired function. The third and final stage is dementia due to AD, when cognition and function are compromised, and is stratified into mild, moderate, or severe (Albert et al., 2011; Sperling et al., 2011). Early detection is key in identifying at-risk individuals who would benefit from future disease-modifying therapies. Biomarkers that accurately reflect disease status and severity may also facilitate the success of future trials by identifying appropriate research participants and helping monitor treatment efficacy. Current widely-accepted biomarkers for AD include cerebrospinal fluid levels of tau and amyloid-beta and amyloid positron emission tomography imaging, but these involve expensive and invasive procedures and are not routinely measured or accessible for patients (El Kadmiri et al., 2018).

In the past few decades, visual biomarkers have become an area of interest with studies showing that AD pathophysiology affects vision-related structures. In 1986, Hinton et al. (1986) were the first to report histological evidence of visual structure degeneration in AD. Post-mortem histological samples showed stark axonal degeneration of optic nerves and retinal ganglion cell loss in AD patients compared to controls (Hinton et al., 1986). Amyloid and tau deposition have also been reported in various vision-related subcortical and cortical structures of the brain. Amyloid plaques and neurofibrillary tangles have been found in the lateral geniculate nucleus (LGN) and primary visual cortex, structures important in relaying and processing of visual information, as well as the superior colliculus and pulvinar nucleus, which are involved in eye movement control (Kusne et al., 2017). Classically, it was thought that AD pathophysiology initially spared these structures, with involvement only in late-stage disease (Albers et al., 2015).

However, more studies suggest that AD pathophysiology may be present in vision-related brain structures earlier on. Neurofibrillary tangles and neuritic plaques have been found in the visual association cortex Brodmann area 19 of patients with preclinical disease and MCI (McKee et al., 2006). More recently, amyloid plaques have been discovered in post-mortem retinas of early AD patients but not controls. Retinal plaques correlated with cerebral and visual cortex amyloid burden and *in vivo* experiments suggest that amyloid plaques in the retina may precede those in the brain (Koronyo-Hamaoui et al., 2011). Moreover, retinal tau pathology in the form of neurofibrillary tangles and hyperphosphorylated tau have been reported in AD patients (Kusne et al., 2017; den Haan et al., 2018). These findings provide histopathological evidence that the visual system may be disrupted much earlier in the AD disease process than previously thought, and thus corroborates the use of visual biomarkers in detecting early disease.

Further, AD patients commonly present with visual symptoms (Katz and Rimmer, 1989; Brewer and Barton, 2014), which is consistent with the fact that roughly half of the cerebral cortex is involved in visual processing (Felleman and Van Essen, 1991). Many studies have also reported significant differences in structural and functional visual measures between AD dementia patients and those with normal cognition (Javaid et al., 2016; Polo et al., 2017; Chan et al., 2019), with some tests capable of detecting MCI (Galetta et al., 2017). Vision is unique because unlike psychometric measures and testing of other senses such as smell, it is often education and culture-invariant and can be more objectively measured. As vision tests are also non-invasive and quick to perform, visual measures have the potential to be practical, cost-effective, and sensitive biomarkers for AD. More studies are now exploring the capacity of visual biomarkers for MCI and preclinical disease detection.

## Search Criteria

A PubMed literature search was conducted to identify all relevant studies within the last 5 years. Our search included Mesh terms or keywords for visual measures such as “visual dysfunction,” “contrast sensitivity,” “color vision,” “color perception,” “optical coherence tomography (OCT),” “eye movements,” “saccades,” “eye tracking,” “anti-saccades,” “pupillary responses, “pupil dilation,” “pupillometry,” “motion processing,” “motion perception,” “visuospatial function” “object identification” “picture naming” “higher visual function” and those related to AD such as “AD,” “cognitive dysfunction,” “MCI,” and “mild neurocognitive disorder.” Only clinical research articles related to AD published from January 2015 to March 2020 and references of these articles were included after a manual curation based on relevance. There are two main reasons for the selection of this time window. Visual markers of AD have been studied for many decades. This topic has also been the focus of review articles published before 2015 (Kirby et al., 2010; Valenti, 2010; Chang et al., 2014; Tzekov and Mullan, 2014). One of our aims was to provide an update by summarizing relevant articles published within the last 5 years. Secondly, modern research diagnostic criteria for MCI and AD dementia, including the use of imaging/cerebrospinal fluid analysis of amyloid-beta and tau, was established in 2011 (Albert et al., 2011; McKhann et al., 2011). Our selected time window ensures that we are including studies using this most recent diagnostic criterion.

## Afferent and Efferent Visual Systems

**Figure 1:**
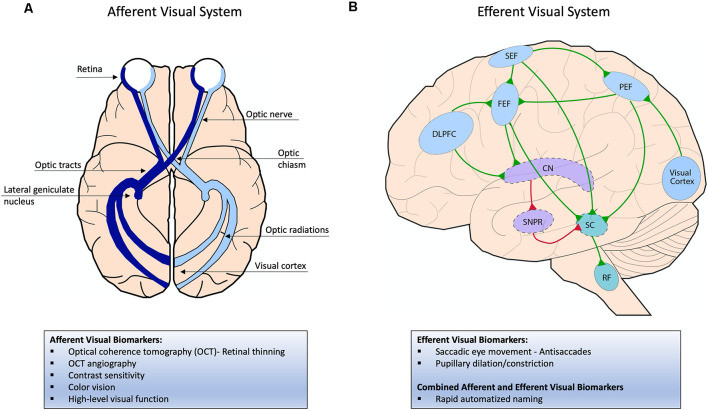
**(A)** Afferent visual system; visual information is initially captured by light-sensitive photoreceptors in the retina and transmitted through the optic nerve and then optic tract, which directly synapses on the lateral geniculate nucleus (LGN) of the thalamus. From the LGN, the optic radiations carry information to the primary visual cortex in the occipital lobe for initial visual processing and then multiple extra-striate cortices for higher-level processing (not pictured here). **(B)** Efferent visual system; To initiate a saccade, excitatory signals from cortical regions such as the frontal, parietal, and supplementary eye fields (FEF, PEF, SEF) are sent to the superior colliculus (SC) in the brainstem, which then projects to the saccade burst generator in the reticular formation. The FEF initiates voluntary and memory-guided saccades, the PEF initiates reflexive saccades, and the SEF initiates saccades that correlate with body movement. In the indirect pathway, the substantia nigra pars reticulata (SNPR) in the basal ganglia sends inhibitory signals to the SC to inhibit a saccade. To override the indirect pathway, the FEF is activated before the saccade generation, which inhibits the SNPR through the caudate nucleus (CN). The dorsolateral prefrontal cortex (DLPFC) helps modulate anti-saccades by inhibiting reflexive saccades and generating subsequent voluntary saccades away from a presented stimulus. Excitatory signals are shown in green and inhibitory signals in red.

The visual system is composed of the afferent and efferent visual systems (Figure 1), which are both affected in AD. The afferent visual pathway is related to the sensory aspect of vision and involves all the structures responsible for receiving, transmitting, and processing visual information. Visual information is initially captured by light-sensitive photoreceptors in the retina and channeled through various synaptic connections to reach the retinal ganglion cells in the inner retina. Retinal ganglion cell axons form the optic nerve and tract and synapse on the LGN of the thalamus. From the LGN, the optic radiations carry information to the primary visual cortex in the occipital lobe for initial visual processing and then multiple extrastriate cortices for higher-level processing including visual recognition and visuospatial processing (Figure 1A; Prasad and Galetta, 2011). The efferent visual pathways are related to the oculomotor part of vision and facilitate eye movements that allow for an in-focus view of objects to capture visual information. Saccades, fast eye movements between various fixation points, are the most common type of eye movement and are generated by a network of cortical and brain stem structures. Excitatory input from cortical regions such as the frontal eye field, parietal eye field, and supplementary eye field and inhibitory input from the basal ganglia feed into the superior colliculus, and signals from the superior colliculus are then sent to the saccade burst generator in the reticular formation to initiate a specific type of saccade. Another important cortical region is the dorsolateral prefrontal cortex (DLPFC), which is particularly important in modulating anti-saccades (Figure 1B; Pierrot-Deseilligny et al., 1995; Girard and Berthoz, 2005).

## Afferent Visual Biomarkers

### Retinal Structure

Advances in ocular imaging modalities now allow for detailed *in vivo* analysis of the optic nerve head and specific layers within the retina, which are affected by AD pathophysiological processes. OCT, a non-invasive imaging technique utilizing infrared light, is capable of unparalleled quantification of retinal thickness and vasculature and has become one of the most studied visual biomarkers in AD.

Despite the presence of negative studies (Pillai et al., 2016; Poroy and Yucel, 2018; Sánchez et al., 2018, 2020; den Haan et al., 2019), there is compelling evidence that significant retinal thinning occurs in AD dementia. AD patients are found to have significant thinning in the peripapillary retinal nerve fiber layer and macular sections containing ganglion cells such as the ganglion cell-inner plexiform layer, ganglion cell layer, and ganglion cell complex (Bambo et al., 2015; Cunha et al., 2016; Garcia-Martin et al., 2016; Ferrari et al., 2017; Kim and Kang, 2019). While the ganglion cell- inner plexiform layer is composed of macular ganglion cell bodies and their dendrites, the ganglion cell layer contains solely the cell bodies, and ganglion cell complex includes the ganglion cell- inner plexiform layer and the macular retinal nerve fiber layer, which represent the ganglion cell axons present on the macula. The peripapillary retinal nerve fiber layer is comprised of ganglion cell axons about to enter the optic nerve. The superior and inferior quadrants of the peripapillary retinal nerve fiber layer often exhibit the most significant reduction (Cunha et al., 2016; Kwon et al., 2017; Kim and Kang, 2019); however, thinning in the temporal region such as the superior temporal and inferior temporal regions have also been reported (Garcia-Martin et al., 2016). Recent meta-analyses support these findings. Mean and quadrant-specific peripapillary retinal nerve fiber layer thickness was significantly reduced in AD patients with the most significant reduction in the superior quadrant (Coppola et al., 2015; Thomson et al., 2015; Chan et al., 2019). A significant reduction of ganglion cell- inner plexiform layer and macular volume or thickness were also seen in AD patients (den Haan et al., 2017; Chan et al., 2019). These structural changes are consistent with post-mortem histology findings of amyloid deposits concentrated in the superior quadrant of the retina and within macular retinal ganglion cells, which may cause anterograde degeneration of the superior quadrant of the optic disc and macular degeneration, respectively (La Morgia et al., 2016; Koronyo et al., 2017). Interestingly, when the retina is examined at pixel resolution, AD participants were found to have a non-uniform distribution of retinal thickening interspersed with thinning compared to controls (Lad et al., 2018; Janez-Escalada et al., 2019). Some studies have also reported macular thickening in moderate stage AD (Salobrar-García et al., 2019). These findings may be a result of inflammatory changes triggered by amyloid deposition or development of granular membranes secondary to retinal gliosis (Zhang et al., 2019a). Although the timeline of these changes is unclear, the proposed dynamic nature of the retina may partially explain negative OCT studies, which examine retinal thickness across pre-set areas. Negative studies may also be explained by non-psychometrically characterized controls (Poroy and Yucel, 2018; den Haan et al., 2019), comparatively younger AD cohorts in the context of no retinal differences in early-onset AD (Pillai et al., 2016; Haan et al., 2019a), and use of different OCT devices (Poroy and Yucel, 2018; Sánchez et al., 2018, 2020).

OCT studies with MCI or subjective cognitive decline are fewer and less congruent (Table 1). The discrepancy in results may be explained by small sample sizes, variable diagnostic criteria, or use of various OCT devices (Oktem et al., 2015; Ferrari et al., 2017; Almeida et al., 2019; Kim and Kang, 2019). While some report no significant differences in the peripapillary retinal nerve fiber layer (Knoll et al., 2016; Lad et al., 2018; Sánchez et al., 2018; Kim and Kang, 2019), others have shown a significant decrease in mean or quadrant-specific peripapillary retinal nerve fiber layer thicknesses in MCI compared to controls (Oktem et al., 2015; Ferrari et al., 2017; Lopez-de-Eguileta et al., 2019; Tao et al., 2019). Even more compelling, a study with amyloid positive- MCI subjects exhibited thinning in the peripapillary retinal nerve fiber layer (Lopez-de-Eguileta et al., 2019). MCI subjects also have reduced thicknesses of the total macula, ganglion cell- inner plexiform layer, and ganglion cell complex, particularly in regions close to the fovea (Gimenez Castejon et al., 2016; Shao et al., 2018; Wu et al., 2018; Almeida et al., 2019). Yet, some studies do not report a difference in either average ganglion cell- inner plexiform layer or macular thickness between MCI and controls (Choi et al., 2016; Pillai et al., 2016; Kwon et al., 2017; Sánchez et al., 2020). Relevant meta-analyses also demonstrate conflicting results. While one meta-analysis found a significant reduction in the peripapillary retinal nerve fiber layer in MCI patients compared to controls (Coppola et al., 2015), a more recent meta-analysis including only spectral-domain-OCT studies found no significant difference in both the peripapillary retinal nerve fiber layer and ganglion cell- inner plexiform layer between these groups (Chan et al., 2019). Interestingly, in these meta-analyses, significant reductions in macular measures including the ganglion cell- inner plexiform layer and macular thickness and volume were only reported in those with Alzheimer’s dementia and not in MCI, while peripapillary retinal nerve fiber layer thinning has been implicated in MCI (den Haan et al., 2017; Chan et al., 2019). This may suggest that the optic nerve head may be affected earlier in disease and serve as a more sensitive site to measure. However, individuals with subjective cognitive decline have been reported to have significantly reduced macular thickness compared to controls (Gimenez Castejon et al., 2016), without having a difference in the peripapillary retinal nerve fiber layer (Santos et al., 2018). In another study, despite no baseline difference in retinal thickness, preclinical AD vs. control participants had significantly decreased macular retinal nerve fiber layer volumes over 27 months (*p* = 0.05; Santos et al., 2018). These results support the macula as the initial site of degeneration, but more studies are needed to determine which layers are affected first.

**Table 1:** Summary of potential afferent visual biomarkers for MCI and preclinical AD.

Citation	*N*	Finding	Avg difference between experimental and control (% change)	*P*-value	Instrument
**Retinal structure**
Oktem et al. (2015)	MCI = 35, HC = 35 participants	↓pRNFL	−9.0 μm (9.8)	<0.001	Cirrus HD-OCT
Ferrari et al. (2017)	MCI = 29, HC = 49 participants	↓pRNFL No diff in macula thickness	−4.7 μm (4.8) −2.6 μm (4.4)	0.033 ns	Fourier-domain OCT
Lopez-de-Eguileta et al. (2019)	MCI = 51, HC = 51 eyes	↓pRNFL	−5.8 μm (5.8)	0.005	Spectralis SD-OCT
Tao et al. (2019)	MCI = 51, HC = 67 participants	↓pRNFL ↓GCC	−8.8 μm (8.2) −5.5 μm (5.6)	<0.01 <0.01	Angiovue SD-OCT
Choi et al. (2016)	MCI = 38, HC = 66 participants	No diff in pRNFL No diff in macula thickness No diff in GC-IPL	−4.7 μm (5.1) −4.6 μm (1.7) −1.5 μm (1.9)	ns ns ns	Cirrus HD-OCT
Knoll et al. (2016)	MCI = 16, HC = 16 eyes	No diff in pRNFL	0.0 μm (0.0)	ns	Spectralis SD-OCT
Pillai et al. (2016)	MCI = 21, HC = 34 participants	No diff in pRNFL No diff in GC-IPL	4.6 μm (5.4) 5.1 μm (6.9)	ns ns	Cirrus HD-OCT
Kwon et al. (2017)	MCI = 16, HC = 16 participants	No diff in pRNFL No diff in macula thickness	1.5 μm (3.3) −0.8 μm (0.3)	ns ns	Cirrus HD-OCT
Lad et al. (2018)	MCI = 30, HC = 36 eyes	No diff in pRNFL	−1.5 μm (1.5)	ns	Spectralis SD-OCT
Sánchez et al., 2018	MCI = 192, HC = 414 participants	No diff in pRNFL	−1.7 μm (1.7)	ns	3D-OCT Maestro
Wu et al. (2018)	MCI = 24, HC = 30 participants	No diff in pRNFL ↓GCIPL	−4.3 μm (4.0) −6.0 μm (6.3)	ns <0.05	Fourier-domain OCT
Almeida et al. (2019)	MCI = 46, HC = 48 eyes	No diff in pRNFL No diff in macula thickness No diff in GC-IPL ↓GCC	−0.3 μm (0.3) −6.8 μm (2.5) −2.0 μm (2.9) −4.1 μm (3.9)	ns ns ns 0.04	Swept source-OCT
Kim and Kang (2019)	MCI = 14, HC = 17 participants	No diff in pRNFL No diff in GC-IPL	−0.8 μm (0.9) 0.2 μm (0.2)	ns ns	Cirrus HD-OCT
Gimenez Castejon et al. (2016)	MCI = 33, HC = 25 participants	↓macula thickness	−15.5 μm (5.6)	<0.05	Cirrus HD-OCT
Shao et al. (2018)	MCI = 24, HC = 21 participants	↓GCIPL	−4.0 μm (6.0)	<0.05	Ultra-high resolution SD-OCT
Sánchez et al. (2020)	MCI = 192, HC = 414 participants	No diff in macula thickness	−4.6 μm (1.7)	ns	3D-OCT Maestro
Santos et al. (2018)	SCD = 15, HC = 41 participants	No diff in pRNFL	1.9 μm (1.9)	ns	Spectralis SD-OCT
Gimenez Castejon et al. (2016)	SCD = 24, HC = 25 participants	↓macula thickness	−13.5 μm (4.9)	<0.05	Cirrus HD-OCT
**Retinal vasculature**
Jiang et al. (2018b)	MCI = 19, HC = 21 participants	↓Parafoveal DVP vessel density No diff parafoveal SVP vessel density	N/A N/A	<0.05 ns	Angioplex OCTA
Wu et al. (2020)	MCI = 32, HC = 33 eyes	↓Parafoveal DVP vessel density ↓Perifoveal DVP vessel density ↑ FAZ No diff in parafoveal SVP vessel density No diff in perifoveal SVP vessel density	−4.2% (8.0) −2.3% (4.4) 0.11 mm^2^ (42.3) −0.1% (0.2) 0.4% (0.7)	<0.001 <0.001 <0.05 ns ns	AngioVue OCTA
Zhang et al. (2019b)	MCI/early AD = 16, HC = 16 participants	↓Parafoveal SVP vessel density No diff in peripapillary RPC vessel density No diff in FAZ	−3.8% (8.6) −2.7% (5.4) −0.02 mm^2^ (8.9)	0.028 ns ns	AngioVue OCTA
Yoon et al. (2019a)	MCI = 72, HC = 254 eyes	No diff in parafoveal SVP vessel density No diff in FAZ	0.09% (0.5) −0.01 mm^2^ (4.0)	ns ns	Angioplex OCTA
Feke et al. (2015)	MCI = 21, HC = 21 participants	↓Venous blood flow ↓Venous blood speed	−3.9 μl/min (19.5) −7.1 mm/s (19.7)	0.009 0.005	Laser doppler retinal blood flow instrument
Jiang et al. (2018a)	MCI = 20, HC = 21 participants	↓venule blood flow rate ↓arteriole blood flow rate	−0.6 nl/s (13.8) −0.7 nl/s (17.0)	<0.05 <0.05	Retinal function imager system
Querques et al. (2019)	MCI = 12, HC = 32 participants	No diff in parafoveal DVP vessel density No diff in parafoveal SVP vessel density ↓retinal reaction amplitude (diff between arterial dilation and constriction)	−0.72% (1.6)	ns ns 0.048	Angioplex OCTA; Dynamic vessel analyzer
O’Bryhim et al. (2018)	Preclinical AD = 14, HC = 16 participants	↑ FAZ	0.09 mm^2^ (32.4)	0.002	Angiovue OCTA
**Contrast sensitivity**
Risacher et al. (2013)	MCI = 28, HC = 29	↓general contrast sensitivity ↑ contrast sensitivity variability	N/A N/A	<0.05 <0.05	FDT 24–2 VF contrast sensitivity test
Risacher et al. (2013)	SCD = 20, HC = 29	No diff in general contrast sensitivity No diff in contrast sensitivity variability	N/A N/A	ns ns	FDT 24–2 VF contrast sensitivity test

*ns, not significant; avg, average; diff, difference; pRNFL, peripapillary retinal nerve fiber layer; GCC, ganglion cell complex; GC-IPL, ganglion cell- inner plexiform layer; DVP, deep vascular plexus; SVP, superficial vascular plexus; RPC, radial peripapillary capillaries; FAZ, foveal avascular zone; CS, contrast sensitivity; MCI, mild cognitive impairment; SCD, subjective cognitive decline; HC, healthy control; HD, high-definition; SD, spectral-domain; OCT, optical coherence tomography; OCTA, optical coherence tomography angiography; FDT, frequency doubling technology; VF, visual field*.

Ultimately, to be a good biomarker, retinal changes should parallel brain processes. Studies have investigated the relationship between retinal measures and brain imaging in individuals who may be healthy or cognitively impaired without dementia. One of these studies shows retinal nerve fiber layer and ganglion cell layer thinning associated with global gray and white-matter volume loss and compromised white-matter microstructure, even when controlling for age, sex, and cardiovascular risk factors (Mutlu et al., 2017). Retinal parameters have also been associated with changes in vision-related brain structures and the temporal lobe. Reduced retinal nerve fiber layer and ganglion cell layer thickness have been associated with lower gray matter density in the visual cortex and lingual gyrus and reduced white matter integrity of the optic radiations on MRI (Méndez-Gómez et al., 2018; Mutlu et al., 2018b; Shi et al., 2020b). It is proposed that this relationship may be explained by either retrograde degeneration from visual cortex damage, anterograde degeneration from retinal ganglion cell loss, or simultaneous retinal and brain changes from AD pathophysiology- which may be more likely as retinal thinning seems to also correlate with other brain changes (Mutlu et al., 2018a). Decreased gray matter volume in the temporal lobes and hippocampal volumes correlated with thinner retinal nerve fiber and ganglion cell- inner plexiform layers (Méndez-Gómez et al., 2018; Shi et al., 2020b). In one study with cognitively normal older adults, thinning of these layers was associated with reduced entorhinal volume in the medial temporal lobe, but not regions associated with aging (bilateral midfrontal cortex) or with later stages of AD (temporoparietal cortex, precuneus, or posterior cingulate region; Casaletto et al., 2017). Since degeneration of the entorhinal cortex is often a hallmark feature of early AD (Albert et al., 2011), perhaps this suggests the potential for the retina to parallel preclinical or prodromal disease. Notably, these retinal measures were not significantly associated with verbal or visual episodic memory performance supporting the notion that changes in the retina and associated medial temporal lobe volumes may precede cognitive change. This is often seen in the case when substantial medial temporal lobe degeneration may occur before any cognitive dysfunction (Jack et al., 2013).

Retinal parameters are also found to correlate with cognition, functioning, and future disease progression. Average and inferior peripapillary retinal nerve fiber layer, ganglion cell- inner plexiform layer, ganglion cell complex, and inner macula thicknesses are reported to moderately correlate with mini-mental status exam scores of AD patients (Cunha et al., 2016). However, not all studies report a correlation (Kim and Kang, 2019). Since mini-mental status exam performance is affected by education level and often not a reliable measure of impairment in mild disease (Arevalo-Rodriguez et al., 2015; Tsoi et al., 2015), Global Deterioration Scale and Clinical Dementia Rating scores are alternatives that better reflect cognition and daily functioning. Using these parameters, one study found that total macula and ganglion cell- inner plexiform layer thicknesses were negatively correlated with the Global Deterioration Scale and Clinical Dementia Rating scores (Kim and Kang, 2019). Clinical Dementia Rating-Sum of box scores, which represent a more granular cognitive scale compared to the normal score, had a negative relationship with ganglion cell- inner plexiform layer thickness and change in this score over 2 years was negatively associated with baseline ganglion cell- inner plexiform layer thickness. Temporal peripapillary retinal nerve fiber layer and average and minimum ganglion cell- inner plexiform layer thicknesses at baseline were also significantly thinner in MCI individuals that progressed to dementia (Choi et al., 2016). Recent longitudinal studies further support retinal parameters as important prognosticators of cognitive decline. One study in roughly 32,000 healthy adults from the United Kingdom showed that those with the thinnest retinal nerve fiber layers were more likely to fail at least one baseline cognitive test and have worse cognition at follow-up 3 years later (Ko et al., 2018). Healthy adults with thinner retinal nerve fiber layer at baseline were also associated with a greater risk of developing AD or any type of dementia after 3–8 years, even with adjustment for cardiovascular risk factors (Mutlu et al., 2018b). Retinal changes perhaps precede cognitive decline and support the potential of retinal parameters as biomarkers even of preclinical disease.

### Retinal Vasculature

Consistent with cerebral vascular dysfunction in AD (Patton et al., 2005), retinal vasculature changes have been reported in AD. With laser doppler and retinal function imager technology, AD patients were found to have decreased blood flow and velocity of retinal veins, venules, and arterioles and reduced retinal tissue perfusion (a function of blood flow and tissue volume) compared to controls. Retinal vein blood flow was also significantly decreased in MCI individuals compared to controls (Table 1, Feke et al., 2015; Jiang et al., 2018a).

The majority of studies investigating retinal microvasculature utilize OCT angiography, a high-resolution, non-invasive imaging technique that can visualize retinal capillaries in specific retinal layers (Kashani et al., 2017). The retinal vasculature of the macula is primarily comprised of two interconnected plexuses: the superficial vascular plexus found in the ganglion cell layer and the deep vascular plexus below the inner nuclear layer. In the fovea, these two vascular plexuses create a region without capillaries known as the foveal avascular zone, which enlarges secondary to retinal ischemia (Conrath et al., 2005). The radial peripapillary capillary plexus is found around the optic nerve head and supplies the retinal nerve fiber layer in this region (Campbell et al., 2017). AD individuals compared to controls are found to have decreased vessel density in both the superficial and deep vascular plexuses mainly in the parafoveal region (2.5 mm from the fovea), but also in outer parts of the macula (Bulut et al., 2018; Jiang et al., 2018b; Yoon et al., 2019a). Parafoveal vessel density has been shown to correlate with mini-mental status exam scores of AD participants. Foveal avascular zone enlargement, often a result of capillary drop out from ischemia, has also been reported in individuals with AD, those with MCI, and those with biomarker-proven preclinical AD (Bulut et al., 2018; O’Bryhim et al., 2018; Wu et al., 2020), but not all have reported significant change from controls (Table 1, Lahme et al., 2018; Yoon et al., 2019a; Zhang et al., 2019b). This discrepancy may be explained by normal variations of this zone due to gender or central retinal thicknesses or unknown confounding factors that may mask a positive finding. Thus, changes in the foveal avascular zone need further investigation (Yoon et al., 2019a). In terms of vascular changes in the optic nerve head, the radial peripapillary capillary plexus was decreased in those with AD dementia, but not those with amnestic MCI (Table 1, Lahme et al., 2018; Zhang et al., 2019b). MCI subjects compared to controls are found to have decreased vessel density specific to the parafoveal deep vascular plexus (Jiang et al., 2018b; Wu et al., 2020). Although, one study reported changes in the parafoveal superficial vascular plexus when considering a group of both MCI and mild AD dementia participants (Zhang et al., 2019b). And another study detected no significant difference between MCI and controls in either plexus, but MCI subjects had a trend of lower density in only the deep vascular plexus (Querques et al., 2019; Zhang et al., 2019b). These results suggest the parafoveal deep vascular plexus may be the more sensitive vascular biomarker. It is proposed that the deep vascular plexus is affected earlier in the disease process because it is composed of smaller vessels than the superficial plexus and therefore more prone to damage from pathophysiologic factors in AD. Perhaps vascular dysfunction starts at the deep vascular plexus closest to the fovea, spreads outward and to other layers of the retina, and then involves the other plexuses. In the presence of vascular changes, some of these studies found corresponding retinal thinning in the peripapillary retinal nerve fiber and ganglion cell- inner plexiform layers (Yoon et al., 2019a), while others did not (Jiang et al., 2018b), suggesting that vascular abnormalities may precede structural change.

These retinal microvascular changes have also been linked to volumetric changes in MRI. Yoon et al. (2019b) performed OCT angiography and volumetric MRI analysis on a small sample of seven MCI individuals and nine AD individuals and found that reduction of the parafoveal vessel and perfusion density correlated with increased inferolateral ventricle volume, a finding secondary to medial temporal lobe atrophy. A few studies have also reported correlations between retinal vasculature parameters and cerebral vascular disease in those with AD. In a study with 48 amyloid-positive AD subjects, vessel density in the perifovea (3–6 mm away from the fovea) was seen to inversely correlate with Fazekas score, a measure of periventricular and subcortical white matter hyperintensity lesions (Haan et al., 2019b). A similar relationship was seen in a group of subjects with amyloid-positive AD, subcortical vascular cognitive impairment, and subjective cognitive decline, where retinal arteriolar fractal dimension negatively correlated with white matter hyperintensity burden (Jung et al., 2019). Interestingly, in a separate study, blood flow density within the superficial vascular plexus in AD subjects was inversely associated with Fazekas scores, but not with amyloid-beta or tau levels in the cerebrospinal fluid (Lahme et al., 2018). These correlations suggest that alterations in retinal microcirculation may parallel that in the brain and cause neurodegeneration. At the same time, there is a possibility that retinal vascular changes are not specific to AD pathology and may reflect comorbid vascular disease. Further studies are needed to assess the specificity of OCT angiography as an AD biomarker.

Despite reports of retinal vascular changes in AD, the underlying pathophysiology remains unclear. However, some histopathological studies have discovered pathogenic forms of amyloid deposits within and along retinal vessels of AD subjects (La Morgia et al., 2016; Koronyo et al., 2017). These findings suggest that vasculature changes may be secondary to amyloid deposition, which may disrupt the blood-retina barrier. This is similar to how cerebral amyloid angiopathy plays a role in blood-brain barrier dysfunction (del Valle et al., 2011). A recent study has explored the integrity of the blood-retina barrier in post-mortem retinas from 56 AD subjects. They report that there is an early and progressive loss of retinal pericytes and vascular platelet-derived growth factor receptor β expression in MCI and AD dementia subjects. Within the brain, platelet-derived growth factor receptor β signaling is proposed to play a role in preserving pericyte count and thus maintaining the blood-brain barrier (Nikolakopoulou et al., 2017). In the study, reduced platelet-derived growth factor receptor β expression was associated with increased amyloid deposits within the retinal vasculature and, more importantly, with cerebral amyloid angiopathy severity, cerebral amyloid plaques, and cognitive status (Shi et al., 2020a). This study suggests that retinal vascular amyloidosis and pericyte loss are present in early AD, mirror brain pathophysiology, and serve as potential visual biomarkers. Given *in vivo* studies using hyperspectral imaging to detect retinal amyloid (Hadoux et al., 2019) and adaptive optics to image retinal pericytes (Schallek et al., 2013), future studies should work towards developing non-invasive technology to visualize these structures in humans. These markers of blood-retina barrier integrity would be promising for early AD detection.

### Contrast Sensitivity

Contrast sensitivity is defined as the ability to differentiate an object from its surroundings and is often considered more sensitive at detecting subclinical visual impairment compared to normal visual acuity (Velten et al., 1999). It is also a measure that correlates with daily function, which makes it a particularly useful measure in AD where deficits in daily function may represent disease progression and increased caretaker needs (Elliott et al., 1990; Dargent-Molina et al., 1996). Different tests that measure low contrast letter acuity (Pelli-Robson), contrast sensitivity across spatial frequencies (CSV-1000E), and contrast sensitivity across a visual field (Frequency doubling technology) have shown deficits in AD. These impairments exist across both low and high spatial frequencies and have been associated with mini-mental status exam and memory test performance (Risacher et al., 2013; Salobrar-García et al., 2019). Interestingly, poor contrast sensitivity correlates with decreased macular thickness and average and superior quadrant retinal nerve fiber layer thickness in AD patients, with a stronger correlation than other functional visual measures such as color vision. A proposed theory is that loss of retinal ganglion cells may at least partially contribute to alterations in visual pathways for contrast sensitivity (Polo et al., 2017). However, the extent of peripheral or central involvement in altered contrast sensitivity remains unclear.

Only one study has investigated contrast sensitivity in individuals with predementia stages of AD. Using frequency doubling technology to assess contrast sensitivity, Risacher et al. (2020) reported significantly reduced general contrast sensitivity and variability of contrast sensitivity across the retina in those with amnestic MCI. This impairment was less pronounced in those with subjective cognitive decline (Table 1, Risacher et al., 2013). These deficits in MCI and subjective cognitive decline have been associated with increased amyloid and tau cortical deposition in temporal, parietal, and occipital lobes, and are significant predictors of amyloid and tau presence on imaging (Risacher et al., 2020). Since contrast sensitivity deficits have been shown to precede the clinical onset of dementia (Ward et al., 2018), these results suggest that contrast sensitivity may be a good biomarker of preclinical and early AD pathophysiology.

### Color Vision

Historically, the ability of color vision tests to reliably differentiate those with and without AD has been controversial with some studies reporting impairment (Pache et al., 2003), while others do not (Wood et al., 1997; Massoud et al., 2002). In recent years, the few new studies examining color vision in AD have reported a difference in test performance from controls. AD patients had impaired chromatic vision based on scores from the Farnsworth D15 and L’Anthony D15, color arrangement tests that detect severe and mild color defects, respectively. Impaired color vision in these participants had a significant, but mild association with retinal thinning, particularly macular thickness. As the parvocellular retinal ganglion cells are responsible for visual pathways that help discern color and pattern (Polo et al., 2017), general retinal ganglion cell loss within the macula may lead to damage to this pathway and subsequent chromatic impairment. However, another study using the Roth 28-Hue test, a similar test to the Farnsworth D15, showed that mild and moderate AD did not exhibit significant dyschromatopsia compared to controls. Yet performance between the groups differed as AD patients had significantly more total errors and non-specific errors in the Tritan (blue spectrum) and deutan (red-green spectrum) regions of the test, with errors correlating inversely with mini-mental status exam scores (Salobrar-García et al., 2019). More studies, preferentially those implementing similar types of chromatic tests, are needed to clarify the degree of chromatic impairment in AD.

### High-Level Visual Function

Higher-order visual processing is also affected by AD. From the primary visual cortex, visual information is proposed to follow one of two streams for higher-level processing. The ventral stream (or “vision for perception” pathway) consists of projections from the primary visual cortex to the occipitotemporal association cortex and is involved in object/form recognition, object discrimination, and facial recognition. The dorsal stream (or “vision for action” pathway) relays information from the primary visual cortex to the parieto-occipital association cortex and determines the spatial layout of a scene, by analyzing motion and spatial relationships between objects and between the body and surrounding visual stimuli (Milner and Goodale, 1995).

In recent years, motion perception deficits have been well studied in AD. A significant finding is that the perception of complex motion is more impaired than simple translational motion in AD and may serve to distinguish AD dementia patients from controls. AD dementia patients performed significantly worse than MCI and similar-aged subjects when detecting rotational motion stimuli displayed with increasing levels of coherence (percentage of background noise disrupting the presented stimuli). In the same study, the authors provided evidence that tasks involving complex motion detection were more effective at differentiating AD severity than those with complex form detection. In addition to motion stimuli, participants were presented with concentric form stimuli of equivalent difficulty. AD dementia patients performed significantly worse on the motion than form task (Porter et al., 2017). In another study, AD dementia patients had significant impairment when detecting motion and direction of optic flow compared to age-matched controls, but direction deficits were less. Conversely, controls had greater impairment of direction than motion detection in the same task, suggesting that complex motion detection rather than directionality may best differentiate AD dementia from normal aging (Liu et al., 2019). This is supported by a study demonstrating that AD dementia patients and controls perform similarly on a horizontal motion discrimination task (Landy et al., 2015). Although there are limited recent studies on motion in MCI, past studies have shown similar deficits in higher-level motion tasks. MCI subjects had significant trouble identifying 3D moving spheres (structure-from-motion) compared to controls, demonstrated by an increased coherence threshold (Table 2, Lemos et al., 2012). In another study, MCI subjects had comparable detection of optic flow motion compared to controls but exhibited significantly delayed EEG responses, particularly in the inferior parietal lobule, during this task. This suggests early dysfunction in high-level dorsal stream processing in AD, which can be mapped to a certain brain region (Table 2, Yamasaki et al., 2012).

**Table 2:** Summary of potential high-level visual function biomarkers for MCI and preclinical AD.

Citation	*N*	Finding	Avg difference between experimental and control (% change)		Visual stimuli/Test
**High-level visual function**
Yamasaki et al. (2012)	MCI = 18, Old HC = 18	↑ P200 ERP latency for optic flow No diff in N170 ERP latency for optic flow No diff in coherence thresholds for optic flow	41.2 ms (17.8) 7.7 ms (3.9) 11.6% (42.6)	<0.001 ns ns	Optic flow motion
Lemos et al. (2012)	MCI = 20, HC = 20	↑ coherence threshold for SFM perception of spheres	N/A	0.035	3D SFM spheres
Lemos et al. (2016)	MCI = 30, HC = 25	↓SFM perception of faces ↓SFM perception of chairs	N/A N/A	0.006 0.016	3D SFM faces/chairs and scrambled objects
Yamasaki et al. (2016)	MCI = 15, Old HC = 16	↑ avg VEP latency in response to faces ↑ N170 VEP latency in response to optic flow No diff in VEP latency in response to low-level ventral (chromatic) stimuli No diff in VEP amplitude in response to low-level dorsal (achromatic) stimuli	16.1 ms (10.5) 32.8 ms (17.6) 6.5 ms (4.8) 0.0 μV (0.0)	0.007 0.001 ns ns	Faces, radial optic flow motion, words (kanji and kana), chromatic stimuli, achromatic stimuli
Gaynor et al. (2019)	MCI = 76, HC = 23	↓performance on object discrimination task was a strong predictor of MCI status	N/A	0.002	Object Recognition and Discrimination Task
Stasenko et al. (2019)	MCI = 852, HC = 3981	↓MINT score in MCI subjects aged 65–75 with 13–15 years of education	−1.7 (5.7)	<0.001	Multilingual Naming Test
Gaynor et al. (2019)	Pre-MCI = 20, HC = 23	↓performance on object discrimination task was a strong predictor of pre-MCI status	N/A	0.02	Object Recognition and Discrimination Task

*ns, not significant; avg, average; diff, difference; MCI, mild cognitive impairment; HC, healthy control; ERP, event-related potential; SFM, structure-from-motion; VEP, visual-evoked potential; MINT, multilingual naming test*.

AD subjects are also reported to have abnormalities in normal, perceptual illusions involving motion. Normal illusions of direction repulsion, motion-induced position shift, and center-surround suppression of large, high-contrast stimuli have all been significantly changed in AD dementia patients compared to controls (Li et al., 2017; Zhuang et al., 2017; Ye et al., 2018). Direction repulsion is the perception that two stimuli are moving away at a greater angle than reality. Motion-induced position shift is the phenomenon where a moving object is perceived to be displaced in the direction of motion, and center-surround suppression is a perceptual phenomenon that causes reduced motion sensitivity of larger stimuli. Increased irregularity on direction repulsion and center-surround suppression tasks correlated with decreased cognitive function as measured by the mini-mental status exam (Li et al., 2017; Zhuang et al., 2017).

The dorsal stream is not the only visual processing pathway impaired in AD. Tests that primarily recruit the ventral stream are also affected. The multilingual naming test, which was recently added to the National Alzheimer’s Coordinating Center’s neuropsychological test battery, is an untimed picture naming test composed of 32 black and white drawings of objects. Performance on this test was significantly different between different stages of AD including MCI vs. controls, MCI vs. mild AD, and mild AD vs. moderate AD. However, good diagnostic accuracy was only achieved between AD dementia and controls (Table 2, Stasenko et al., 2019).

Many tasks involving both visual processing streams are also affected in AD. AD dementia patients performed significantly worse than controls on the Hooper Visual Organization Test, which requires timely identification of 30 line drawings of objects that have been fragmented into pieces (Mitolo et al., 2016). This task requires mental reorganization of the parts for identification of a whole object and has been shown to stimulate dorsal and ventral stream cortical regions on functional MRI (Moritz et al., 2004). MCI subjects have similar trouble with mental rotation and object identification (Table 2). The object recognition and discrimination task is a test wherein each trial, participants are shown four presented stimuli [three of which are the same object (non-target), and one that is different (target)], and asked to identify the target stimulus as quickly and accurately as possible. During “difficult” trials, a visuospatial component is added where non-target stimuli are rotated. Performance on difficult trials was able to distinguish controls from AD dementia, amnestic MCI, and even pre-MCI (defined as clinical dementia rating score of 0.5 with no memory impairment on psychometric testing or clinical dementia rating of 0 with memory impairment on testing; Gaynor et al., 2019). These results are consistent with early AD pathology in the form of tau deposition affecting the perirhinal cortex, which is crucial for feature integration for object identification and discrimination (Devlin and Price, 2007; Sone et al., 2017). Detection of moving faces and chairs (structure-from-motion perception) was also worse in MCI patients compared to controls, but less so for chairs (Table 2). On functional MRI, performance on this task was positively correlated with the cortical thickness of occipital lobe regions and ventral fusiform areas involved in visual face processing (Lemos et al., 2016). These studies taken together suggest widespread cortical dysfunction involving both streams in AD.

Interestingly, impairment of the ventral and dorsal streams in MCI is selective for high-level pathways. Low-level ventral and dorsal stream pathways are thought to exist between the retina and the primary visual cortex. Low-level ventral pathways process high spatial resolution, low temporal resolution, low contrast sensitivity, and color sensitivity, while low-level dorsal pathways process low spatial resolution, high temporal resolution, high contrast sensitivity, and color insensitivity. In a small study, MCI patients had significantly prolonged visual evoked potentials in response to higher-level ventral (faces), and dorsal (optic flow motion) stimuli, but not to lower-level stimuli (Table 2). In contrast, similar-aged controls had relatively preserved VEPs in response to higher-level, but not lower-level ventral and dorsal stimuli (Yamasaki et al., 2016). Amyloid-beta deposits, the earliest indicator of AD pathology, is thought to first originate in the cortex, and proceed to subcortical regions of the brain (Hardy and Selkoe, 2002). Although primary sensory cortices such as the primary visual cortex are initially spared by this deposition, the temporal, occipital, and parietal lobe can be involved earlier during this process (Braak and Braak, 1991). Amyloid-beta deposition in these areas may disrupt neuronal connections in high-level visual processing areas, and thus cause for impairment in prodromal disease. This theory is supported by findings of gray matter atrophy in three dorsal-stream-related visual cortices in late MCI and extensive atrophy of cortices related to dorsal and ventral stream processing in AD dementia individuals (Deng et al., 2016).

## Efferent Biomarkers

### Saccadic Eye Movement

In the last few decades, saccadic eye movement abnormalities have become one of the most common types of oculomotor dysfunction documented in AD. With high-quality video-based eye trackers, these eye movements are also easily captured. Saccades are quick eye movements that allow for the eyes to fixate on various spatial locations, placing the object of interest onto the fovea, the region of the retina responsible for the highest visual acuity. The majority of AD studies have focused on prosaccades, eye movements towards a presented stimulus, and antisaccades, eye movements away from a stimulus. AD patients are noted to have longer latencies in initiating prosaccades and antisaccades and have increased errors on antisaccade tasks with more errors left uncorrected compared to controls (Crawford et al., 2005, 2013; Yang et al., 2011, 2013; Noiret et al., 2018). Errors on the antisaccade task occur when the participant makes an eye movement toward rather than away from a presented stimulus. Self-corrected errors occur when a participant makes an error, but quickly corrects by looking away from the presented stimulus. Other eye movements that are affected in AD include smooth pursuit, the action of continuously following and maintaining gaze on a moving target, and microsaccades, which are less than one-degree shifts in the gaze that occur naturally during fixation. AD patients often have more non-horizontal, or oblique, microsaccades compared to controls (Kapoula et al., 2014), and during smooth pursuits tasks, AD patients struggle to initiate saccades and often make either compensatory saccades to keep up with the target or premature, anticipatory saccades leading the target (Hutton et al., 1984; Garbutt et al., 2008).

Studies within the last 5 years support previous eye movement findings in Alzheimer’s dementia patients and provide more evidence for saccadic eye movements as biomarkers for earlier-stage disease. Most studies that include MCI include participants with amnestic MCI, a subgroup characterized by memory impairment and most likely to progress to AD dementia (Fischer et al., 2007). Unlike studies with AD dementia participants, prosaccade and antisaccade latencies do not reliably distinguish between amnestic MCI and controls (Kahana Levy et al., 2018). However, reduced saccadic accuracy during prosaccade and antisaccade tasks has been reported in amnestic MCI patients compared to controls (Table 3, Holden et al., 2018; Chehrehnegar et al., 2019), and previously reported in AD (Yang et al., 2011). Saccadic accuracy refers to the amplitude of the eye movement compared to that of the target stimulus. However, the most robust changes in oculomotor function seen in amnestic MCI seem to be in antisaccade tasks. A recent meta-analysis reported that 87% of the 20 studies examined showed AD or MCI patients with increased antisaccade error rates compared to controls (Kahana Levy et al., 2018). Increased antisaccade errors in amnestic MCI participants compared to controls were also found in a study using a commercial, automated eye video tracker, with antisaccade errors inversely correlating with mini-mental status exam score. Number of self-corrected errors differentiated mild AD dementia, MCI, and cognitively normal individuals from each other (Table 3, Holden et al., 2018).

**Table 3:** Summary of potential efferent and combined visual biomarkers for MCI and preclinical AD.

Citation	*N*	Finding	Avg difference between experimental and control (% change)	*P*-value	Instrument
**Saccadic Eye Movement**
Holden et al. (2018)	MCI = 29, HC = 27	↑ proportion of antisaccade errors ↓gain for prosaccades No diff in antisaccade latency No diff in prosaccade latency	22.6% (93.0) −0.04 (4.3) 33.8 ms (12.9) N/A	<0.001 0.03 ns ns	EyeBRAIN tracker
Wilcockson et al. (2019)	MCI = 42, HC = 92	↑ proportion of antisaccade errors ↑ antisaccade latency	20.0% (200.0) 81.0 ms (24.0)	<0.0005 <0.0005	EyeLink eye tracker
Chehrehnegar et al. (2019)	MCI = 49, HC = 59	↓the first gain for prosaccades ↓the first gain for antisaccades No diff in antisaccade latency No diff in prosaccade latency	−0.1 (11.4) −0.2 (20.6) 14.2 (3.7) 13.3 (3.9)	<0.01 <0.01 ns ns	SMI RED system eye tracker
**Pupillometry**
Granholm et al. (2017)	MCI = 53, HC = 793	↑ Task-evoked pupil dilation during 3-digit span test ↑ Task-evoked pupil dilation during 6-digit span test	0.08 mm (N/A) 0.07 mm (N/A)	<0.001 0.026	NeurOptics PLR-200 pupillometer
Frost et al. (2017)	Preclinical AD = 38, HC = 77	During pupil flash response: ↓maximum constriction velocity No diff in mean constriction velocity No diff in mean constriction acceleration No diff in the mean amplitude of constriction	−0.4 mm/s (9.6) −0.3 mm/s (9.6) −2.9 mm/s^2^ (8.8) −0.1 mm (8.4)	0.021 ns ns ns	NeurOptics VIP-200 pupillometer
Van Stavern et al. (2019)	Preclinical AD = 24, HC = 33	During pupil flash response: No diff in mean constriction velocity No diff in percent constriction No diff in latency of constriction	N/A N/A N/A	ns ns ns	NeurOptics PLR-200 pupillometer
**Combined Afferent and Efferent Tests**
Galetta et al. (2017)	MCI = 39, HC = 135	Longer (worse) rapid number naming	10.5 s (17.6)	0.002	King-Devick test

*ns, not significant; avg, average; diff, difference; MCI, mild cognitive impairment; HC, healthy control*.

Most interestingly, Wilcockson et al. (2019) were the first group to study saccadic eye movement within MCI subtypes. They found that the antisaccade error rate is significantly higher in those with amnestic MCI compared to those with non-amnestic MCI and was negatively associated with memory score as measured by the free and cued selective reminding test free recall (Table 3, Wilcockson et al., 2019). Different subtypes of MCI are often determined by thorough neuropsychological testing with amnestic MCI defined as an impairment of memory alone or with other cognitive domains and non-amnestic MCI defined as an impairment of at least one non-memory cognitive domain (Kelley and Petersen, 2007). Results from this study are important because they introduce an easier test that could potentially differentiate MCI subtypes. As amnestic MCI is associated with a higher risk of progression to AD dementia, performance on the antisaccade task may help identify those truly at risk of Alzheimer’s dementia.

A theory for why antisaccades are more sensitive in detecting earlier AD than prosaccades is that antisaccades require more higher-level executive processing. Performing an antisaccade task requires controlled inhibition of a reflexive saccade towards a visual stimulus and then intact working memory to make a saccade away from the stimulus, which are both functions that are associated with the DLPFC, an area found to be active during antisaccade tasks (Hutton and Ettinger, 2006). Consistent with this explanation are results that show the frequency of antisaccade errors correlating with various neuropsychological tests that relate to executive function but with strongest correlation to the Stroop test, which measures inhibitory control (Heuer et al., 2013; Holden et al., 2018). Also, spatial working memory was highly correlated to the frequency of uncorrected antisaccade errors (Crawford et al., 2013). Spatial working memory is thought to be important in actively maintaining the task goal when presented with a visual stimulus in the antisaccade task. Slight lapses in working memory would allow for loss of inhibition of the presented stimulus and could result in uncorrected errors (Unsworth et al., 2004). Meanwhile, performance on prosaccades requires automatic processing and saccade initiation (Peltsch et al., 2014), which may require less cognitive burden and thus be relatively spared in early-stage disease.

### Pupillometry

Task-evoked pupillary dilation is a promising visual biomarker in AD. Pupil dilation has been of interest because it is linked to activity in the locus coeruleus, which undergoes degenerative changes in early AD (Grudzien et al., 2007; Braak et al., 2011). The relationship between the pupil and locus coeruleus is particularly evident during cognitive tasks, where pupil dilation and neuronal activation in the locus coeruleus increase in parallel with cognitive effort. A functional MRI study has also shown an association between locus coeruleus-generated cortical activity and impaired task-related pupil dilation (Elman et al., 2017). The locus coeruleus is important in AD because studies suggest that this region may be the earliest site of tau pathology, and tau neurofibrillary tangles and volume loss in the locus coeruleus are associated with AD duration and severity (Bondareff et al., 1987; Grudzien et al., 2007; Braak and Del Tredici, 2011). During cognitive tasks, pupil dilation is a measure of compensatory cognitive effort. Thus, someone with reduced cognitive ability at baseline would exhibit increased pupil dilation, until the task at hand requires a cognitive load that exceeds baseline ability and compensatory effort, in which pupil size would decrease and performance declines. In one study, single-domain amnestic MCI participants exhibited greater pupil dilation than single-domain non-amnestic MCI and controls when asked to perform digit-span recall tasks with lower cognitive load. However, task performance did not differ between groups (Table 3, Granholm et al., 2017). These results suggest that pupillary changes may capture subtle cognitive abnormalities not yet manifest in cognitive performance measures and that pupil dilation can differentiate between MCI subtypes. Interestingly, impaired task-evoked pupil dilation in cognitively normal adults correlated with a higher risk of progression to MCI (Kremen et al., 2019). This supports the use of task-evoked pupillary measures in identifying at-risk individuals for AD. However, more studies are needed to explore their potential as a visual biomarker.

Light-induced pupillary responses have also been studied in AD. Pupillary size and response are controlled by opposing sympathetic and parasympathetic systems. In the presence of light, pupillary constriction is driven by the parasympathetic or cholinergic system, which requires acetylcholine to induce iris sphincter contraction (Spector, 1990). This response can be measured by a pupillometer with a white flash stimulus and is called the pupil flash response. Past studies show that AD dementia patients have abnormal, less reactive pupil flash responses (Fotiou et al., 2000, 2007; Granholm et al., 2003). It is thought that impairment in this response may be due to cholinergic deficiency in AD patients. More recent literature supports these findings. AD dementia patients were found to have significantly reduced pupil constriction acceleration, velocity, and amplitude and significantly increased latencies to constriction compared to controls (Fotiou et al., 2015; Frost et al., 2017). These pupillary responses were correlated with mini-mental status exam and Wechsler Memory Scale scores (Fotiou et al., 2015). Only two studies have explored light-induced pupillary responses in preclinical AD. In a small study comparing biomarker-positive preclinical AD to controls, there was no significant difference in pupil constriction velocity, percent constriction, or constriction latency between groups (Van Stavern et al., 2019). Another study found reduced maximum constriction velocity in preclinical AD compared to controls but no other differences in pupil flash response (Frost et al., 2017). These findings suggest that abnormalities in light-induced pupil responses are found in later stage AD, but are smaller and less measurable in preclinical disease.

## Combined Visual Tests

Performance on vision-based tests that incorporate both the afferent and efferent visual system is also compromised in AD. The King-Devick Test is a timed rapid number naming test that requires participants to read out loud numbers arranged in different configurations and spacing. Performance on this test requires intact lower-order and higher-order visual processing, visual tracking, and saccadic eye movements. In one study, the King-Devick test was shown to be a highly sensitive screening test for both MCI and AD dementia, as it was able to differentiate these groups from controls (Table 3, Galetta et al., 2017). Other vision-based rapid automatizing naming tests exist but have not yet been tested in those with AD. The Mobile Universal Lexicon Evaluation System, a rapid picture naming test, however, has been shown to reliably detect concussion and other neurodegenerative diseases such as Parkinson’s disease and multiple sclerosis (Akhand et al., 2018; Seay et al., 2018; Conway et al., 2020). These tests are unique because they assess various dimensions of vision, but also those for cognitive processes such as attention, concentration, processing speed, and language. Since all these areas can be affected by AD, these tests may serve as a more robust screening tool.

## Discussion

There is growing evidence that visual measures can differentiate early-stage AD, particularly MCI, from those without disease. These visual measures and potential biomarkers fall under both the afferent and efferent visual systems with some correlating with cognition, brain neurodegeneration, and AD pathophysiology. It is important to note that in recent years, literature in this area has focused predominantly on the afferent limb of the visual system. There has been a particular focus on examining visual pathway structure in the retina with OCT and OCT angiography techniques. This may be due in part to the novelty of some of these techniques and also their accessibility, as OCT has been successfully integrated into ophthalmic clinical practice. This trend highlights where the field is going, but also the need for further investigation of efferent markers, especially those that may show more promise than afferent markers. Further, although we discuss many promising visual measures in this review, there are still many important questions that need to be addressed.

A strong biomarker is one that has high sensitivity. Currently, it remains unclear which visual measures are most sensitive at detecting the earliest disease given the small number of studies with MCI and preclinical disease (subjective cognitive decline or no symptoms), if any. To best assess sensitivity, future studies should focus on individuals with MCI or preclinical disease. These individuals should ideally be well-characterized with accepted AD biomarkers to guarantee AD etiology, especially since MCI can have multiple etiologies. Controls should also be psychometrically characterized as normal to best detect differences from those with early disease, as well as have their amyloid and tau status documented. Currently, few existing studies have biomarker-positive participants and some use healthy volunteers as controls or mini-mental status exam score cutoffs, which do not reliably separate those that are normal from MCI (Trzepacz et al., 2015). Investigating more vision-based cognitive tests may also increase sensitivity because these tests involve more neural circuits that may be impaired.

Another attribute of a good biomarker is specificity. A known challenge of visual biomarkers is that visual changes can be shared between AD and other dementias or age-related neurodegenerative and ophthalmic diseases. It is hard to assess which visual biomarkers are most specific because few studies directly compare disease entities. However, some visual measures have been shown to differentiate between etiologies. For example, retinal vasculature is impaired in different regions in glaucoma vs. AD and increased horizontal saccade latency differentiates AD from frontotemporal dementia (Boxer et al., 2012; Zabel et al., 2019). More studies are needed to evaluate individual biomarker specificity. Although machine learning requires big data, implementing these techniques on quantitative forms of data such as retinal structural and vasculature data may help differentiate AD from other common etiologies. Machine learning techniques have been successful at differentiating individuals with glaucomatous damage from those without using OCT-derived parameters (Burgansky-Eliash et al., 2005). Multimodal visual testing may also increase specificity by creating distinct visual profiles for AD.

Based on current literature, there are a few visual measures that seem most promising as strong, practical biomarkers. Saccadic eye movement, particularly errors on the antisaccade task can differentiate amnestic MCI from controls, and even memory from non-memory impaired MCI subtypes. Performance on a combination of prosaccade and antisaccade tasks can further distinguish AD from Lewy body dementia, Parkinson’s disease dementia, and frontotemporal dementia (Mosimann et al., 2005; Antoniades and Kennard, 2015). Eye movements can be easily captured by a commercial, automated video-tracking system and with proper software, eye movements can be auto-coded and analyzed. A relatively new biomarker, which deserves future exploration, is task-evoked pupillary dilation, which differentiates MCI subtypes and has been associated with future risk of MCI. This test may be especially sensitive and specific to AD because performance parallels the activity of the locus coeruleus, which is an initial site of subcortical tau deposition in early-stage AD (Braak et al., 2011). All these visual tests often require access to a single, commercially sold machine, which is less expensive than current techniques to obtain AD biomarkers. Rapid automatized naming tests may also be sensitive and cost-effective. These tests simultaneously assess multiple aspects of vision and cognition such as visual processing, saccadic eye movement, object-related memory, color vision, and language, which may be impaired in AD. Usually presented in a physical or digital format, they do not require any expensive equipment. These tests are not specific but may be combined with another test to enhance specificity. Similarly, tests evaluating motion perception, visuospatial function, and object identification/discrimination may be particularly sensitive for early disease, as these often involve multiple cortical regions that may be affected by amyloid deposition. Retinal thinning has been the most studied biomarker in recent years and has been associated with cognition, brain changes, and future dementia. However, lack of specificity and the ability to consistently identify early-stage disease make it difficult to use as a diagnostic biomarker.

Despite the number of studies in this field, many unexplored areas remain. With most studies focused on typical AD, there is a paucity of studies examining visual biomarkers in atypical presentations including logopenic primary progressive aphasia, a frontal variant of AD, and posterior cortical atrophy. Clinically, these forms of AD overlap with other non-AD dementias and often rely on AD biomarkers for a more definitive diagnosis (Wolk, 2013). Since all have similar distributions of cerebral amyloid pathology compared to typical AD (Wolk, 2013), visual measures may also be affected and serve as diagnostic markers in these presentations. Another area to explore is visual biomarkers in early-onset AD characterized by autosomal dominant mutations in amyloid precursor protein and presenilin genes and late-onset AD associated with the apolipoprotein epsilon 4 allele. These genes play crucial roles in amyloid plaque accumulation in AD (Selkoe and Hardy, 2016). Since amyloid deposition tomography differs in early and late-onset AD (Cho et al., 2013), these gene mutations may be associated with differential amyloid deposition in the brain and in vision-related structures. It would be interesting to investigate if visual biomarkers can also detect early onset-AD and if these various gene mutations influence the type of visual impairment experienced.

The ability of visual biomarkers to differentiate between dementia types and MCI types has also been largely unexplored. However, there is much clinical value in finding a visual measure that can distinguish between dementia types, particularly AD from Lewy body dementia, which is often hard to clinically differentiate in early disease (McKeith et al., 2005). Lewy body dementia subjects have more impairment in retinal structure, contrast sensitivity, saccadic latencies, and color vision than their AD counterparts (Mosimann et al., 2005; Moreno-Ramos et al., 2013; Oishi et al., 2018). In particular, color vision impairment is a strong predictor of Lewy body dementia more so than in AD where color vision defects are less frequent and perhaps less pronounced (Postuma et al., 2015; Matar et al., 2019). Regarding high-level visual processing, Lewy body dementia patients also exhibit worse motion discrimination and performance on tests requiring spatial processing and object identification (Landy et al., 2015; Mitolo et al., 2016). Future studies should assess for differences in color test performance, motion perception, and visuospatial function between these two groups of patients. Antisaccade performance should also be assessed in these subject groups since this task has been successful in differentiating amnestic MCI individuals (most likely to progress to AD) from non-amnestic MCI (most likely to progress to Lewy body or frontotemporal dementia; Kelley and Petersen, 2007).

Finally, longitudinal studies examining multiple biomarkers together should be performed to establish temporality and causality. Retinal structure, vasculature, visual function, and brain changes are interlinked, but thus far have only been shown to correlate at different stages of disease. Longitudinal assessment would clarify visual changes that occur earliest in the disease process and identify those that can be used to assess disease progression. Longitudinal studies would also assess whether changes in visual measures over time can serve as biomarkers. AD participants are reported to have a faster decline in retinal thickness compared to controls (Trebbastoni et al., 2016; Santos et al., 2018). The degree of decline in visual measures may confer differential risk of disease progression and should be further investigated.

Through this review, we highlight potential afferent and efferent visual biomarkers of AD and explore their ability to detect prodromal and preclinical disease. Although more studies are needed, visual measures are promising, objective, practical, and sensitive markers of AD.

## Author Contributions

All authors contributed to the article and approved the submitted version. SW was responsible for literature review, manuscript drafting, and table and figure design. AM and LB were involved in manuscript editing. SW, AM, and LB were involved in revisions. AM and LB were involved in providing oversight of this project.

## Conflict of Interest

The authors declare that the research was conducted in the absence of any commercial or financial relationships that could be construed as a potential conflict of interest. The reviewer Z-LL declared a shared affiliation, with no collaboration, with the authors to the handling editor at the time of review.
